# A Comparative Study of Early Afterdepolarization-Mediated Fibrillation in Two Mathematical Models for Human Ventricular Cells

**DOI:** 10.1371/journal.pone.0130632

**Published:** 2015-06-30

**Authors:** Soling Zimik, Nele Vandersickel, Alok Ranjan Nayak, Alexander V. Panfilov, Rahul Pandit

**Affiliations:** 1 Department of Physics, Centre for Condensed Matter Theory, Indian Institute of Science, Bangalore, Karnataka, India; 2 Department of Physics and Astronomy, Ghent University, Ghent, Belgium; 3 Robert Bosch Centre for Cyber Physical Systems, Indian Institute of Science, Bangalore, Karnataka, India; 4 Jawaharlal Nehru Centre for Advanced Scientific Research, Bangalore, Karnataka, India; 5 Moscow Institute of Physics and Technology (State University), Dolgoprudny, Moscow Region, Russia; University of Oxford, UNITED KINGDOM

## Abstract

Early afterdepolarizations (EADs), which are abnormal oscillations of the membrane potential at the plateau phase of an action potential, are implicated in the development of cardiac arrhythmias like Torsade de Pointes. We carry out extensive numerical simulations of the TP06 and ORd mathematical models for human ventricular cells with EADs. We investigate the different regimes in both these models, namely, the parameter regimes where they exhibit (1) a normal action potential (AP) with no EADs, (2) an AP with EADs, and (3) an AP with EADs that does not go back to the resting potential. We also study the dependence of EADs on the rate of at which we pace a cell, with the specific goal of elucidating EADs that are induced by slow or fast rate pacing. In our simulations in two- and three-dimensional domains, in the presence of EADs, we find the following wave types: (A) waves driven by the fast sodium current and the L-type calcium current (Na-Ca-mediated waves); (B) waves driven only by the L-type calcium current (Ca-mediated waves); (C) phase waves, which are pseudo-travelling waves. Furthermore, we compare the wave patterns of the various wave-types (Na-Ca-mediated, Ca-mediated, and phase waves) in both these models. We find that the two models produce qualitatively similar results in terms of exhibiting Na-Ca-mediated wave patterns that are more chaotic than those for the Ca-mediated and phase waves. However, there are quantitative differences in the wave patterns of each wave type. The Na-Ca-mediated waves in the ORd model show short-lived spirals but the TP06 model does not. The TP06 model supports more Ca-mediated spirals than those in the ORd model, and the TP06 model exhibits more phase-wave patterns than does the ORd model.

## Introduction

The heart is an electro-mechanical pump whose contractile activity is mediated by electrical waves generated periodically in the Sino Atrial Node. Abnormal electrical excitations in the heart can disrupt the normal propagation of these electrical waves and cause life-threatening arrhythmias like ventricular fibrillation (VF). Arrhythmias can have different underlying causes. One particular group of arrhythmias can be connected to special excitations of cardiac cells, called early afterdepolarizations (EADs). Early afterdepolarizations are anomalous oscillations of the membrane potential of a cell at the repolarizing phase of the action potential (AP); they are commonly seen in patients with an acquired or congenital long-QT syndrome [1–3], and subjects with heart failure [4–7]. Early afterdepolarizations can be induced through the administration of pharmacological drugs [8–12], or because of oxdidative stress [13–15] and fibrosis [16, 17]. Early afterdepolarizations are pro-arrhythmic because of their potential ability to induce dispersed refractory periods in cardiac tissue and to facilitate the formation of premature triggers, which are the two vital conditions for the precipitation of arrhythmias. Early afterdepolarizations are often linked to the potentially lethal arrhythmias like Torsade de Pointes; however, the basic mechanisms still remain incompletely understood of how single-cell abnormalities lead to whole-heart arrhythmias.

Many experimental [1, 18–20] and computational [20–22] studies have been performed to investigate the ionic mechanisms of EADs in single-cell studies. Early afterdepolarizations are induced when the repolarization reserve (RR) is reduced to such an extent that a reversal of the normal repolarization (depolarization) takes place. This can be obtained by increasing the inward currents or reducing the outward currents, or both, of the cells. So, for example, a cell can be made susceptible to EADs by increasing the conductances of the inward L-type calcium current (I_CaL_) and decreasing the conductances of outward currents like the delayed rectifier potassium currents [(I_Kr_ (rapid component) and I_Ks_ (slow component)] [23, 24]. Early afterdepolarizations can also occur because of pathological calcium dynamics in the cell, e.g., calcium overloading in the Sarcoplasmic Reticulum (SR), an organelle inside the cell that serves as the calcium store, which leads to spontaneous calcium release and ultimately the reactivation of I_CaL_ mediated by the enhancement of the Na/Ca exchanger current (I_NaCa_) [18, 19, 25, 26]. Although many computational and experimental studies have investigated EAD in single cells, very little is known about how EADs give rise to arrhythmias in 2D tissue and the whole heart. The solution to these challenging problems requires the use of multi-scale mathematical modeling [27–30]. In Ref. [31], a computational study was done to investigate how clumps of cells, eliciting EADs in synchrony, give rise to triggered activities, which can disturb any prevailing course of wave propagation and induce electrical-wave turbulence. This phenomenon of local synchronization of the abnormal depolarizations of EAD cells is implicated in the formation of premature ventricular complexes (PVCs) [32, 33]. A systematic investigation of the relation between single-cell EADs and 2D cardiac arrhythmias, using a ventricular human-cell model due to Ten Tusscher and Panfilov (TP06 [34]), has been presented in Ref. [23]. This study has identified the following three different types of fibrillation, by reducing RR via an increase of the conductance of I_CaL_ and decrease of that of I_Kr_: (A) The first type of fibrillation displays waves that are mediated by both I_Na_ and I_CaL_ [35] and consist of chaotic unstable spiral waves. (B) The second type comprises only waves mediated by I_CaL_ and exhibits stable and unstable spirals. (C) The third type yields phase waves, which are pseudo-travelling waves that we discuss in detail later.

Given the role that EADs can play in the precipitation of cardiac arrhythmias, it is important to carry out a study that compares the effects of EADs on electrical-wave dynamics in different realistic models for cardiac tissue. Such a comparative study can help us identify those effects that are common to such models and those that are not. The latter must be studied especially carefully (e.g., in experiments) to make sure that they are not artifacts of a given mathematical model for cardiac tissue. With this motivation in mind, we carry out a detailed comparative study of EADs in two state-of-the-art models for human ventricular tissue, namely, the TP06 model mentioned above and the O’Hara-Rudy model [36], which we refer to as the ORd model. The TP06 and ORd models are similar insofar as they have the same number of major ionic currents, such as the Na current (I_Na_), I_CaL_, I_Kr_, I_Ks_, etc. These models differ in the number of minor ionic currents, e.g., the plateau and background currents, they use, and in the ways in which they model some of the major and minor ionic currents. These differences are briefly discussed in the Methods section below.

It is important to compare the single-cell behaviors of the two models. Therefore we investigate the different regimes in both these models, namely, the parameter regimes where they exhibit (1) a normal AP with no EADs, (2) an AP with EADs, and (3) an AP (with EADs) that does not go back to the resting potential. Furthermore, we study the dependence of EADs on the rate of pacing a cell, with the specific goal of elucidating EADs that are induced by slow [10, 19, 37–39] or fast rate pacing [19, 40–42]. We find that, with the reduction of RR by enhancing I_CaL_ and reducing I_Kr_, the ORd model displays slow-rate-dependent EADs and the TP06 model shows fast-rate-dependent EADs. Despite the difference in the rate dependence of EADs at the single-cell level, both these models give rise to the same three types of waves, namely, Na-Ca-mediated, Ca-mediated, and phase waves. However, there are quantitative differences in the wave patterns of each wave type, which we discuss in detail below.

The remaining part of this paper is organized as follows. The Section entitled Materials and Methods describes the models we use and the numerical methods we employ to study them. The Section entitled Results contains our results, both from single-cell and tissue-level simulations. The Section entitled Discussions is devoted to a discussion of our results in the context of earlier numerical and experimental studies.

## Materials and Methods

### Description of the two models

The two models, TP06 and ORd, have the same number of major ionic currents but differ in the number of minor current-carrying components, like the plateau and background currents. The ORd model incorporates a K^+^ background current that is not incorporated in the TP06 model; the TP06 model has a plateau K^+^ current that is not included in the ORd model. The ORd model leads in the number of minor ionic currents by incorporating additional K^+^ and Na^+^ currents through the L-type Ca^2+^ channel. In total, the ORd model has 14 ionic currents and the TP06 model has 12 ionic currents. Although the two models do not differ much in terms of the ionic currents, the modelling of some of the ionic currents of the same type are different in the two models. In the ORd model I_Na_ is a sum of a fast and a slow component, whereas the TP06 model considers only a fast component. The ORd model accomodates two components of Na^+^/Ca^2+^ exchanger currents, a subspace component, and a component from the bulk myoplasm. In contrast, the TP06 model just incorporates a single Na^+^/Ca^2+^ exchanger current from the myoplasm. An important protein kinase, which plays a crucial role in calcium-signaling process, and which also modulates the kinetics of certain ion channels through phosphorylation, is the Ca^2+^/Calmodulin dependent kinase CaMKII. The ORd model takes into account the role of CaMKII in its calcium dynamics, and also the effect of CaMKII on the kinetics of various ion channels, like the I_CaL_ channel (L-type), I_Na_ channel, and I_to_ channel. The TP06 model does not include such detailed calcium dynamics with CaMKII.

In a single cell the membrane potential (V_m_) is governed by the ordinary differential equation (ODE)

$$\begin{array}{c} \frac{\partial V_{m}}{\partial t}=-\frac{I_{\text{model}}}{C}, \end{array}$$ (1)

where *C* is the capacitance of the cell; *I*_model_ is the sum of all the ionic currents in each model. We give this below for both the models.

$$\begin{array}{c} I_{\text{ORd}}=I_{\text{Na}}+I_{\text{to}}+I_{\text{CaL}}+I_{\text{CaNa}}+I_{\text{CaK}}+I_{\text{Kr}}+I_{\text{Ks}}+I_{K1}+I_{\text{NaCa}}+I_{\text{NaK}}+I_{\text{Nab}}+I_{\text{Cab}}+I_{\text{Kb}}+I_{\text{pCa}}; \end{array}$$ (2)

$$\begin{array}{c} I_{\text{TP}06}=I_{\text{Na}}+I_{\text{to}}+I_{\text{CaL}}+I_{\text{Kr}}+I_{\text{Ks}}+I_{K1}+I_{\text{NaCa}}+I_{\text{NaK}}+I_{\text{bNa}}+I_{\text{bCa}}+I_{\text{pK}}+I_{\text{pCa}}. \end{array}$$ (3)

A glossary of all the ionic currents is given in Table 1.

**Table 1:** Table of currents.

ORd		TP06	
I_Na_	Na^+^ current	I_Na_	Na^+^ current
	(sum of a fast and a slow component)		(a single fast component)
I_to_	transient outward K^+^ current	I_to_	transient outward K^+^ current
I_CaL_	Ca^2+^ current	I_CaL_	Ca^2+^ current
	through the L-type Ca^2+^ channel		through the L-type Ca^2+^ channel
I_Kr_	rapid delayed rectifier K^+^ current	I_Kr_	rapid delayed rectifier K^+^ current
I_Ks_	slow delayed rectifier K^+^ current	I_Ks_	slow delayed rectifier K^+^ current
I_K1_	inward rectifier K^+^ current	I_K1_	inward rectifier K^+^ current
I_NaCa_	Na^+^/Ca^2+^ exchange current	I_NaCa_	Na^+^/Ca^2+^ exchange current
	(sum of a subspace and a myoplasmic component)		(single myoplasmic component)
I_NaK_	Na^+^/K^+^ ATPase current	I_NaK_	Na^+^/K^+^ ATPase current
I_Nab_	Na^+^ background current	I_bNa_	Na^+^ background current
I_Cab_	Ca^2+^ background current	I_bCa_	Ca^2+^ background current
I_pCa_	sarcolemmal Ca^2+^ pump current	I_pCa_	Ca^2+^ plateau current
I_Kb_	K^+^ background current	I_pK_	K^+^ plateau current
I_CaNa_	Na^+^ current through the L-type Ca^2+^ channel		
I_CaK_	K^+^ current through the L-type Ca^2+^ channel		

The various ionic currents incorporated in the ORd and TP06 models are tabulated above. The symbols used for the currents follow Refs. [36] and [34] for the ORd and TP06 models, respectively.

The spatio-temporal evolution of the membrane potential (V_m_) in a tissue is governed by a reaction-diffusion equation, which is a partial-differential equation (PDE):

$$\begin{array}{c} \frac{\partial V_{m}}{\partial t}+\frac{I_{\text{model}}}{C}=D\nabla^{2}V_{m}, \end{array}$$ (4)

where *D* is the diffusion constant.

For details of the algebraic equations modeling the ionic currents of the models, we refer the reader to Refs. [36] and [34] for ORd and TP06 models, respectively. It is important to note that we have made a few modifications in the case of the TP06 model. In particular, to obtain EADs in the TP06 model, the time constant of the f-gate of the L-type Ca current has been decreased twofold [23]. As this results in some shortening of the AP, we have increased the value of the conductance of the calcium current by a factor of 2.

### Numerical Methods

**Fig 1:**
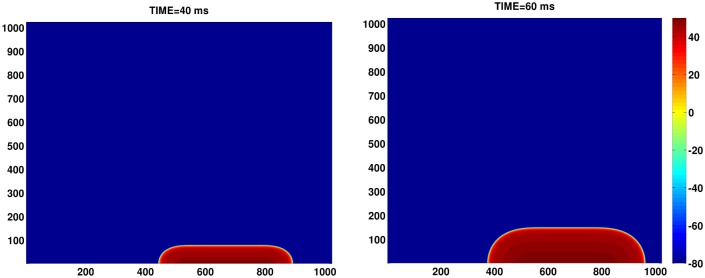
The assymetric-pulsing protocol used for our tissue simulations. Pseudocolor plots of the membrane potential V_m_ illustrating the growth of a wave generated by an asymmetric pulse, stimulated over a region of 0.12 × 6 cm^2^ (6 × 300 grid points) at the lower boundary of the domain. The strength of the stimulus current is −150 *μ*A/*μ*F, applied for a duration of 3 ms.

We solve the ODE for V_m_ for a single cell and the ODEs for the gating variables of the ionic currents with a forward-Euler method. For solving the PDE (2), we use the forward-Euler method for time marching with a five-point stencil for the Laplacian in two dimensions (2D) and seven-point stencil in three dimensions (3D). We set *D* = 0.003 cm^2^/msec and *D* = 0.00154 cm^2^/msec for the ORd and TP06 models, respectively. The temporal and spatial resolutions in the two models are as follows: ORd: *δx* = 0.02 cm, *δt* = 0.02 msec; TP06: *δx* = 0.025 cm, *δt* = 0.02 msec. The above combinations of diffusion constants, and time and space steps, gives a conduction velocity (CV) of 65 cm/sec, in the ORd model, and 67 cm/sec, in the TP06 model. In our 2D simulations we use a domain size of 1024 × 1024 grid points, for the ORd model, and 1000 × 1000 grid points, for the TP06 model, which translate into physical sizes of 20.48 × 20.48cm^2^ and 25 × 25cm^2^, respectively. And for our 3D simulations we use the same number of grid points in the *x* − *y* plane and, in the *z* direction, we add 10 grid points for the ORd model, and 8 grid points for the TP06 model to give a thickness of 2 mm, a typical thickness of the human endocardium [43]. All our 2D and 3D simulations are carried out for a duration of 10 seconds. We use two protocols for initiating spiral waves. The first is the conventional S1-S2 cross-field protocol in which we apply a stimulus (S1) of strength −150 *μ*A/*μ*F for 3 ms to the bottom edge, of our 2D domain, or the left face, of our 3D simulation domain. As a result we obtain a propagating plane wave. We then apply the second (S2) stimulus, of the same strength and duration as S1, from the bottom boundary to almost half of the domain, i.e., 0 cm ≤ *y* ≤ 10 cm for 2D, and 0 cm ≤ *y* ≤ 10 cm and 0 mm ≤ *z* ≤ 2 mm for 3D. This S1-S2 procedure generates spiral and scroll waves in our 2D and 3D domains [44, 45]. For the second protocol, we use asymmetric pulsing in which an external stimulus is applied over a small region (0.12 × 6 cm^2^ in 2D and 0.12 × 6 × 0.2 cm^3^ in 3D) on the lower boundary of the domain as shown in Fig 1. The strength and duration of the stimulus of this asymmetric pulse are comparable to their counterparts for the above S1-S2 cross-field protocol.

## Results

We begin with a comparison of our single-cell results for the ORd and TP06 models. We then present the results of our simulations in 2D and 3D domains for these models.

### Single Cell

#### Stability diagrams from single-cell simulations

**Fig 2:**
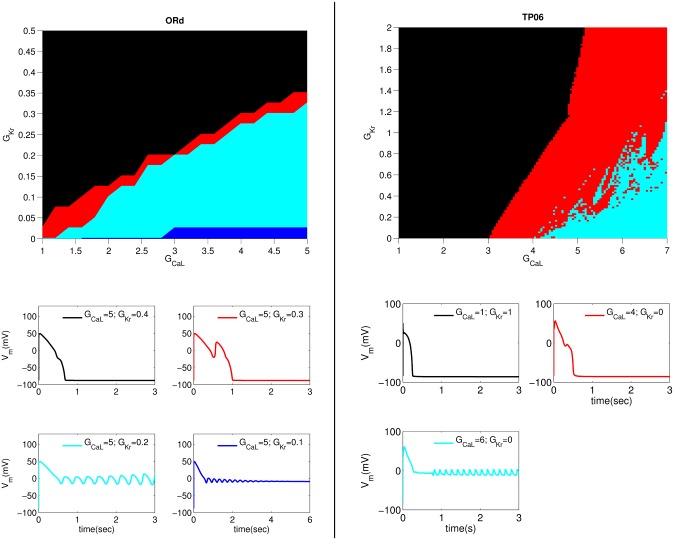
Stability diagrams for different types of APs in the G_CaL_-G_Kr_ parameter space. In the top panel, black, red, cyan and blue indicate, respectively, regions of the following AP types: (1) a normal AP; (2) an AP with one or more EADs that returns to the resting state; (3) an AP that is in a triggered (oscillatory) state; (4) an AP, which, after few oscillations, relaxes towards a new higher resting potential. The ORd stability diagram is presented in the top-left panel and the TP06 one in the top-right panel. The cell is paced at PCL = 1000ms and the states of the APs are decided by considering the final AP after 50 pulses. Here the values of G_CaL_ and G_Kr_ indicate the multiples of their control values. Hence, G_CaL_ = 5 and G_Kr_ = 0.4 denote, respectively, that we use a value of G_CaL_ (G_Kr_) that is 5 (0.4) times its control value. The bottom panel elucidates the APs of types (1)-(4) shown, respectively, by black, red, cyan, and blue curves for representative values of G_CaL_ and G_Kr_ for ORd (bottom-left) and TP06 (bottom-right) models.

Early afterdepolarizations can be induced by decreasing the repolarization reserve (RR) of a cell. In Fig 2 (top panel) we present stability diagrams, in the G_CaL_ and G_Kr_ plane, for different types of APs, which are seen when the cell is paced with a pacing cycle length (PCL) of 1000ms, in TP06 and ORd models. Here and henceforth we normalize the values of these conductances by their control values; e.g., 0.2 on the vertical axis of Fig 2 (top panel) denotes that the value of G_Kr_ is 0.2 of its control value given in the original model. In Fig 2 (bottom panel) we show, for both these models, plots of APs that can occur in the stability regions; the colors of these AP plots are the same as those for the corresponding stability regions. The first type (1) is a normal AP, shown in black in Fig 2 (bottom panel). The second type (2) is shown in red, and represents an AP with one or multiple EADs. The third type (3), shown in cyan, is the AP with non-decaying EAD oscillations and does not return to the normal resting potential. A fourth type (4), shown in blue, is similar to type (3) and does not repolarize to the resting state. However, the oscillations in the AP of type (4) decay with time and V_m_ finally relaxes to a higher value than the normal resting value. We have obtained this type of AP only in the ORd model, within the parameter regime of Fig 2 (however, in other parameter regimes we can get type (4) APs in the TP06 model). By comparing both the stability diagrams in Fig 2 we find that, to obtain EADs, we must reduce RR, by changing G_CaL_ and G_Kr_, much more in the TP06 model than in the ORd model. Early afterdepolarizations are induced in the ORd model by a reduction of G_kr_ to 15% of its control value. In contrast, even if we block I_Kr_ fully, no EADs can be induced in the TP06 model.

#### Rate dependence of EADs

**Fig 3:**
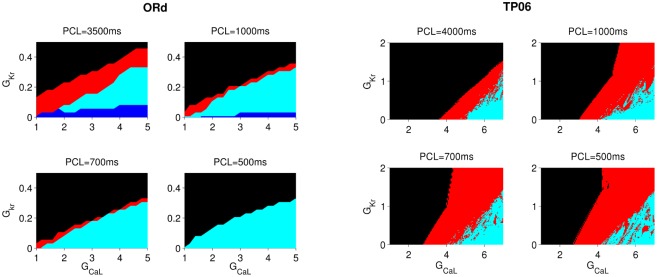
PCL dependence of EAD regions in the ORd and TP06 models. The dependence on PCL of the EAD region for the ORd (left panel) and TP06 (right panel) models. Note that the red region, EAD associated with AP type-(2), shrinks in the ORd model as PCL decreases; by contrast, it expands in the TP06 model as PCL decreases.

The formation of EADs depends on the rate at which we pace the cell [19]. We investigate this rate dependence for both ORd and TP06 models by plotting in Fig 3 stability diagrams, such as those in Fig 2, for different values of PCL. We apply 50 pulses to stimulate the cell at each different value of PCL and then decide on the type of AP by examining the last AP. From Fig 3 we see that, in the ORd model, as PCL decreases the stability regions of APs of types (2) and (4) decrease. The red, the type-(2) region and the type-(3) (cyan) and type-(4) (blue) regions below it all exhibit EADs, but the type-(1) (black) region does not. In the ORd model, the black region grows at the expense of the regions with EADs as we decrease PCL (from 3500ms to 500ms in Fig 3); therefore, this model exhibits slow-rate-dependent EADs, i.e., EADs appear more prominently at large values of PCL (slow rate pacing) than at small values of PCL (fast rate pacing). By contrast, in the TP06 model, the red, type-(2) region expands at the expense of the type-(1), black region as we decrease PCL, i.e., the EADs here show a fast rate dependence. The mechanisms of the different rate dependence of EADs will be investigated elsewhere. Here we concentrate on the effects of such EADs on wave propagation in tissues in both ORd and TP06 models.

### Tissue (2D)

In this subsection we present a detailed and systematic study of our 2D simulations. We elaborate on the types of waves observed in the ORd and TP06 models and also compare the wave patterns of the different types of fibrillation.

#### Types of spiral waves

**Fig 4:**
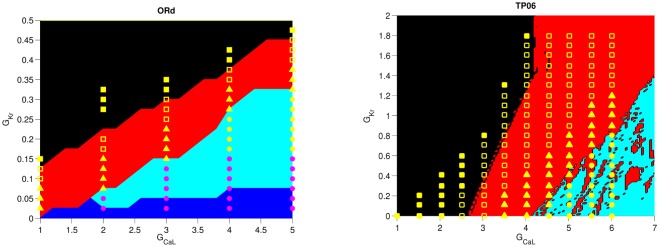
Points at which different types of waves are observed in the G_CaL_-G_Kr_ parameter space. Enlarged versions of two of the stability diagrams of Fig 3 (PCL = 3500ms for the ORd model and PCL = 500ms for the TP06 model) showing the types of waves, which occur at different values in the G_CaL_-G_Kr_ plane, by the following symbols: yellow and magenta circles (phase waves); yellow-filled triangles (Ca-mediated waves); open squares (Na-Ca-mediated waves); yellow-filled squares (waves mediated by I_Na_).

Three types of wave patterns, in the presence of EADs, have been found in a 2D-tissue study by Vandersickel, *et al.* [23]. We compare these three wave patterns in the ORd and the TP06 model in the subsequent sections. Here, we give an overview of the parameter regimes where these three wave types appear. We have summarized the results for both models by superimposing different symbols on the single-cell stability diagrams in Fig 4. The yellow-filled squares indicate the region where no EAD activity is observed, and the spiral waves initiated at these points are mediated by I_Na_. Now, if we reduce the RR gradually, say by keeping G_CaL_ fixed and reducing G_Kr_, triggered excitations because of EADs start appearing in the medium, and the waves begin to be mediated not only by I_Na_ but also by I_CaL_ [35]. First, waves mediated both by I_Na_ and I_CaL_ are formed; we call this type Na-Ca-mediated waves. In Fig 4 the open squares indicate points at which we obtain these Na-Ca mediated waves. On reducing the RR further, the waves in the tissue can be solely driven by I_CaL_, with I_Na_ almost absent or too low to drive a wavefront; we call these Ca-mediated waves. And finally, we have also found phase waves in the ORd model, which are pseudo-travelling waves that are not obstructed by impenetrable obstacles. These phase waves occur in regions of the parameter space marked by yellow filled circles in Fig 4; the magenta-filled circles indicate points at which we find phase waves initially but the amplitude of the waves decay with time and the medium relaxes to a refractory steady state. From Fig 4 we see that, in both ORd and TP06 models, as we reduce RR, we progress from Na-Ca-mediated waves, to Ca-mediated waves, and finally to phase waves.

**Fig 5:**
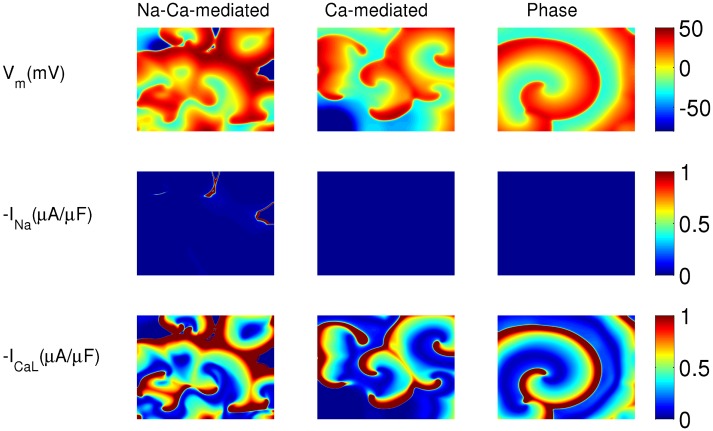
Pseudocolor plots depicting the three types of spiral-fibrillations in the ORd model: Pseudocolor plots of V_m_ (top row), I_Na_ (middle row), and I_CaL_ (bottom row) illustrating Na-Ca-mediated (left column), Ca-mediated (middle column), and phase (right column) waves. The parameter sets are as follows. Na-Ca-mediated wave: G_CaL_ = 4 and G_kr_ = 0.37; Ca-mediated wave: G_CaL_ = 4 and G_kr_ = 0.275; phase wave: G_CaL_ = 4 and G_kr_ = 0.2.

**Fig 6:**
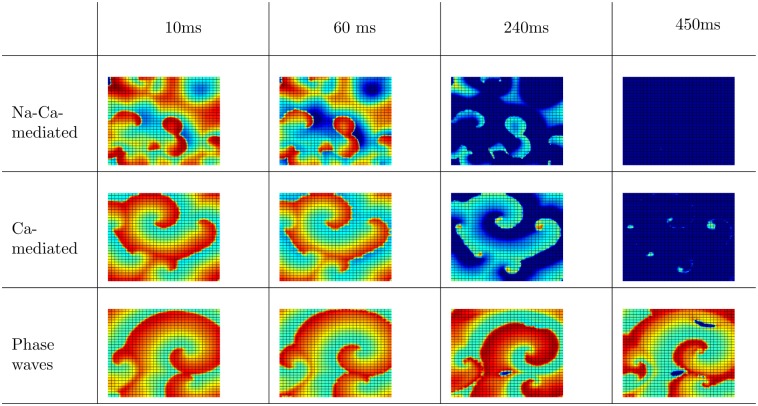
Illustration of the time evolution of the three wave-types in the ORd model with obstacles: Pseudocolor plots of V_m_, at representative times between 10ms and 450ms, illustrating the elimination of Na-Ca-mediated and Ca-mediated waves by a mesh that divides the simulation domain into squares of 32 × 32 grid points. We set the diffusion constant to zero at the boundaries of the squares. Note that phase waves (row 3) are not eliminated by this mesh. The parameter sets are as follows. Na-Ca-mediated wave: G_CaL_ = 4 and G_kr_ = 0.37; Ca-mediated wave: G_CaL_ = 2 and G_kr_ = 0.175; phase wave: G_CaL_ = 5 and G_kr_ = 0.225.

The types of waves, because of EADs, that can occur in the TP06 model have been characterized in Ref. [23]. In Fig 5, and we show a similar characterization for the ORd model. Fig 5 shows the three different wave types and the corresponding I_Na_ and I_CaL_ currents (see also S1 Video). The top, middle, and bottom panels of Fig 5 show pseudocolor plots of V_m_, −I_Na_, and −I_CaL_, respectively, for the three wave-types for the ORd model. We see that, in the Na-Ca-mediated type, I_Na_ is active in some, but not all, wavefronts. In the Ca-mediated type and phase waves, only the I_CaL_ current is active, and there is hardly any I_Na_ current. Note that all the wave-types are initially driven by I_Na_; in the course of time I_Na_ becomes almost zero in the case of Ca-mediated and phase waves, but it continues to drive the Na-mediated waves for the 10s duration of our simulation (in S1 Video, I_Na_ becomes zero in the case of Ca-mediated and phase waves after 9s). Fig 6 shows the time evolution of these three wave-types in the presence of a mesh of impenetrable obstacles; see also S2 Video. The mesh divides the whole domain into small squares of 32 × 32 grid points, which are electrically decoupled from each other; we achieve this by setting the diffusion constant, *D*, to zero on the boundaries of the squares. As can be seen from Fig 6, the Na-Ca-mediated and Ca-mediated waves are eliminated by the presence of the mesh, but the phase waves persist. The Na-Ca-mediated and Ca-mediated waves are conventional waves that propagates in a medium through the process of diffusion of ions in synergy with the excitability of the medium. In these two wave-types, the diffusion current-flux is the source of the stimulus that excites the unexcited neighboring cells at the wavefronts; any form of decoupling of the cells, say, by insertion of impenetrable obstacles, blocks the propagation of these waves. Phase waves, on the other hand, are not obstructed by any impenetrable obstacles. Phase waves occur when the cells in the medium support APs of type (3) or type (4) (Fig 2), i.e., APs with oscillations that do not repolarize back to the normal resting potential. Phase waves are not real travelling-waves like the Na-Ca-mediated and Ca-mediated waves, but they appear because of the presence of a timing gradient in the medium [46]. As phase waves do not need a diffusion current for their propagation, the insertion of impenetrable obstacles does not block the ‘propagation’ of the wave-front.

#### Na-Ca-mediated waves

**Fig 7:**
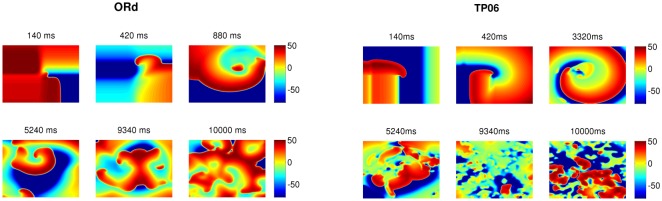
Typical wave patterns of Na-Ca-mediated waves in the ORd and TP06 models. The left and right panels show the time evolution of typical wave patterns of Na-Ca-mediated wave-types in the ORd and TP06 models, respectively. The parameter sets for the two models are as follows. ORd: G_CaL_ = 4 and G_kr_ = 0.37; TP06: G_CaL_ = 3 and G_kr_ = 0.5.

**Fig 8:**
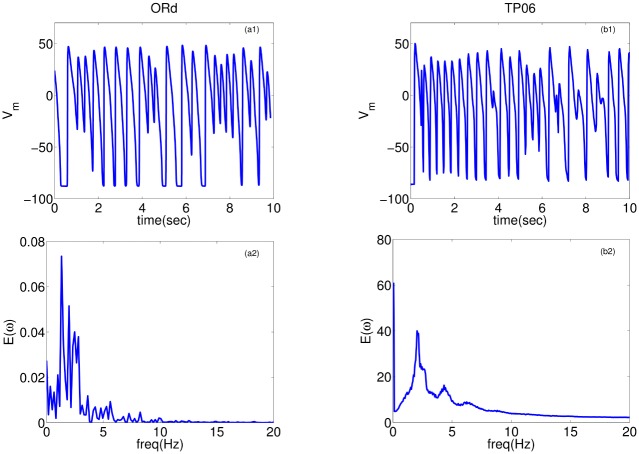
Time-series and power spectra of Na-Ca-mediated waves: Plots of V_m_ versus time for the ORd (a1) and TP06 (b1) models. The second row, (a2) ORd and (b2) TP06, shows the averaged power spectra of the time-series; these spectra are obtained by averaging the spectra of time-series of V_m_ over 2500 grid points. Parameter sets: ORd: G_CaL_ = 4 and G_kr_ = 0.37; TP06: G_CaL_ = 3.5 and G_kr_ = 0.8.

The wave patterns of the Na-Ca-mediated type are slightly different in the two models. The ORd model supports spirals that are short-lived, whereas the TP06 model does not produce any spiral waves that last for one complete rotation. Fig 7 shows the time evolution of this wave-type in both these models for the representative parameter sets provided in Fig 7 (a corresponding Video is provided below; S3 Video). After initiating the spiral, with the S1-S2 cross-field protocol, the spirals in the ORd and TP06 models show the first triggered activity because of EADs in the examples presented at times 880ms and 3320ms, respectively, after the application of the S2 stimulus. This initial triggered activity, which introduces functional heterogeneity in the medium, further exacerbates the instability of the spiral and leads to more triggered excitations and eventually induces wave turbulence in the medium. The waves in the ORd model continue to exhibit short-lived spirals, whereas the waves in the TP06 model do not exhibit spirals that sustain themselves for one complete rotation (see also S3 Video). Typical time series recordings of V_m_, for this wave-type in both the models, from a point, and the power spectra (E(*ω*)) averaged over 2500 grid points are shown in Fig 8. Both the models show a wide spectrum of frequencies. However, the frequency of the strongest peak in E(*ω*) is related approximately to the inverse of the spacing between successive peaks in the time series of V_m_.

#### Ca-mediated waves

**Fig 9:**
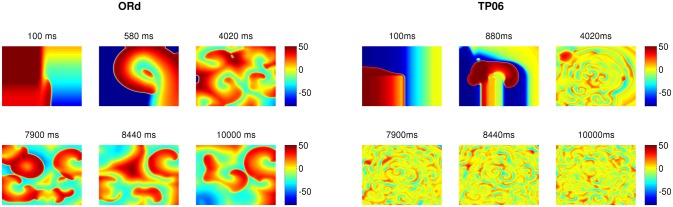
Typical wave patterns of Ca-mediated waves in the ORd and TP06 models. The left and right panels show the time evoultion of typical wave patterns of the Ca-mediated wave-type in the ORd and TP06 models, respectively. The parameter sets for the two models are as follows. ORd: G_CaL_ = 4 and G_kr_ = 0.275; TP06: G_CaL_ = 3.5 and G_kr_ = 0.

**Fig 10:**
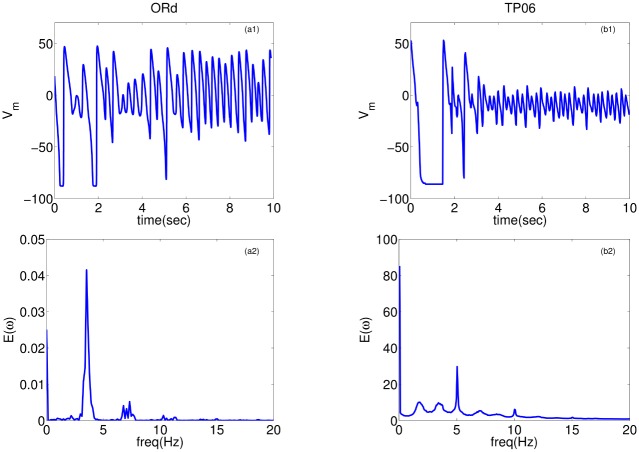
Time-series and power spectra of Ca-mediated waves: Plots of V_m_ versus time for the ORd (a1) and TP06 (b1) models and their averaged power spectrums (a2) and (b2), respectively. Parameter sets: ORd: G_CaL_ = 4 and G_kr_ = 0.275; TP06: G_cal_ = 5 and G_kr_ = 0.6

These waves are initially driven by I_Na_, but the fraction of wavefronts mediated by I_Na_ decreases in the course of time and eventually the waves are totally mediated by I_CaL_, within the 10s of the duration of simulation (see S1 Video). The TP06 model supports more spirals than does the ORd model, because the spiral arms in the former are narrower than in the latter, perhaps because the duration of the secondary oscillations (see Fig 2) is larger in the ORd model than in the TP06 model. Fig 9 shows the time evolution of a spiral, initiated by the S1-S2 cross-field protocol, into multiple spirals in both these models (see also S4 Video). The time-series of V_m_ and the averaged power spectrum, obtained as in the earlier subsection (**Na-Ca-mediated waves**), is shown in Fig 10. Both these models show a prominent peak in the E(*ω*), which indicates the frequency of the small oscillations in the time-series of V_m_ that oscillates about a voltage higher than −50 mV. This peak frequency also corresponds to the frequency of rotation of the spirals in the medium. In Fig 10, the value of this peak frequency is 3.5 Hz in the ORd model, and 5.18 Hz in the TP06 model; these values increase with an increase of G_CaL_. The wave patterns of this wave-type in both the models are similar, to the extent that there are multiple stable spirals (Fig 9), and E(*ω*) exhibits a single prominent peak corresponding to the rotation frequency of the spirals.

#### Phase waves

**Fig 11:**
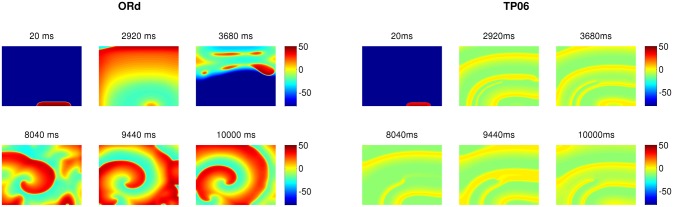
Typical wave patterns of phase waves in the ORd and TP06 models. The left and right panels show the time evolution of phase waves in the ORd and TP06 models, respectively, when an assymetric pulse is applied. The parameter sets for the two models are as follows. ORd: G_CaL_ = 4 and G_kr_ = 0.2; TP06: G_CaL_ = 5 and G_kr_ = 0.

**Fig 12:**
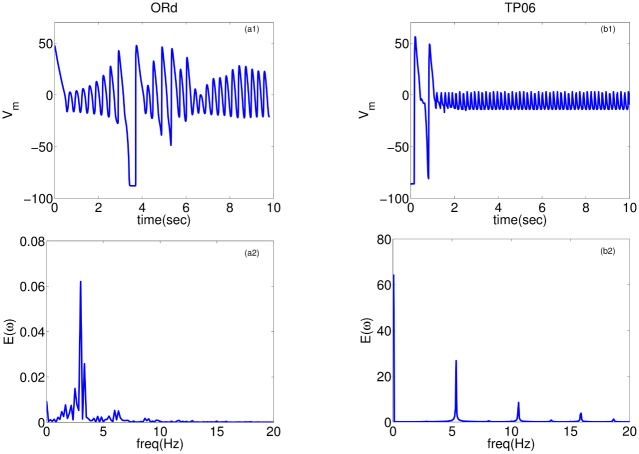
Time-series and power spectra of phase waves: Plots of V_m_ versus time for the ORd (a1) and TP06 (b1) models and their averaged power spectra E(*ω*) (a2) and (b2), respectively. Parameter sets: ORd: G_CaL_ = 4 and G_kr_ = 0.2; TP06: G_CaL_ = 5 and G_kr_ = 0.1

**Fig 13:**
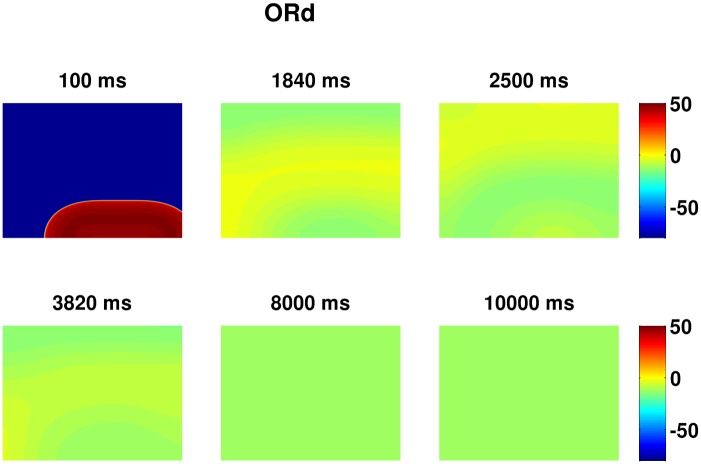
Refractory steady state. Psedocolor plots of V_m_ illustrating the progression of the medium into a refractory steady state when G_Kr_ is very low such that the repolarizing power of the medium is obliterated. Parameter set: G_CaL_ = 4 and G_kr_ = 0.15.

In the ORd model, the phase waves usually evolve into spirals or periodic wavetrains when we apply a single, asymmetric pulse. On the other hand, this wave-type shows a variety of patterns in the TP06 model [23], which include various unusual manifestations; e.g., in addition to spirals, point sources, and lines of point sources have been shown to emerge. We show the time-evolution of this wave-type, for both these models, in Fig 11 for specific parameter sets (also see S5 Video). In the ORd model, after some initial trains of waves emerge spontaneously from the point of application of the initial stimulus, excitations, triggered by EADs, lead to backfiring of waves (see left panel at 3680 ms in Fig 11 and S5 Video), and these backfired waves eventually evolve into spirals of phase waves. In the TP06 model, however, the trains of waves are not disrupted by any triggered excitations during their propagation and the wave trains continue to progress undisturbed for more than 20s. The time series and the averaged power spectrum of this wave-type are shown in Fig 12. In both the models, E(*ω*) shows a prominent peak. The peak frequency is the frequency of the spirals in the ORd model, and in the TP06 model, it is the frequency of the periodic wave trains. Phase-wave patterns with uninterrupted wave-trains, similar to those in the TP06 model, can also be produced in the ORd model as we reduce the value of G_Kr_; Fig 13 shows one such example. However, in the ORd model, the amplitude of the waves decay with time and the medium eventually relaxes to a steady state with a potential ≃ −10mV. Once the medium attain this steady state, it becomes completely refractory and does not allow the conduction of any external stimulus. This happens when the cells in the medium exhibit the AP type (4) (Fig 2) where G_Kr_ is reduced to a level that the repolarizing power of the cell is obliterated. The points where this steady state occurrs in the G_CaL_-G_Kr_ parameter space are marked by magenta-colored circles in Fig 4.

### Tissue (3D)

**Fig 14:**
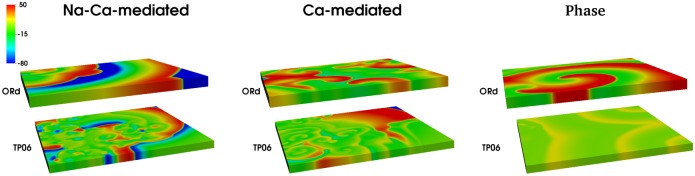
Pseudocolor plots showing V_m_ on the surfaces of our 3D domain illustrating the wave patterns of the three wave-types in ORd and TP06 models. The leftmost set of figures represents the Na-Ca-mediated type; the middle represents the Ca-mediated type; the rightmost represents the phase waves. The parameter sets are as follows. Na-Ca-mediated: (ORd) G_CaL_ = 4 and G_Kr_ = 0.37; (TP06)G_CaL_ = 3 and G_Kr_ = 0. Ca-mediated: (ORd) G_CaL_ = 4 and G_Kr_ = 0.275; (TP06)G_CaL_ = 4 and G_Kr_ = 0. Phase waves: (ORd) G_CaL_ = 4 and G_Kr_ = 0.2; (TP06) G_CaL_ = 6.8 and G_Kr_ = 0.1.

It is instructive to see how the Na-Ca-mediated, Ca-mediated, and phase waves manifest themselves in three-dimensional (3D) simulation domains. So, we perform simulations for representative parameter sets of each wave type, and the wave patterns of the three wave types are shown in Fig 14. For the Na-Ca- and Ca-mediated waves (left and right panels in Fig 14, respectively) we use the S1-S2 cross-field protocol to initiate scroll waves. In the ORd model, short-lived scroll waves are observed in the Na-Ca-mediated case, as in our 2D simulations where short-lived spirals are observed for this wave type. By contrast, in the TP06 model, the scroll waves do not sustain themselves for a period of one complete rotation (see S6 Video). For the Ca-mediated type, both the ORd and the TP06 models produce stable scroll waves (see S7 Video). To initiate phase waves (right panel of Fig 14) we use an assymetric pulse, as in 2D, for both ORd and TP06 models. Again, the ORd model produces phase waves, which are stable scroll waves (as in 2D where stable spirals are obtained); the TP06 model produces periodic wave trains from the opposite end of the boundary where the stimulus is applied (see S8 Video).

## Discussions

In this paper, we have investigated the differences and similarities in EAD generation and 2D pattern formation because of EADs in the ORd and TP06 models. In the first part of our paper, we have compared the single-cell behaviors of the two models in terms of generating EADs. Although these models have different underlying descriptions of the currents, they display qualitatively similar behaviors when we gradually reduce the repolarization reserve (RR). Four types of APs are found as we reduce the RR: (1) a standard AP with no EADs, (2) an AP with finite EAD oscillations, (3) an AP with sustained EAD oscillations, and (4) an AP with decaying EAD oscillations, in which V_m_ relaxes to a new, high resting potential (see Fig 2). We conjecture that this behavior of the cell exhibiting APs of different morphologies as the RR is reduced, is universal for all mathematical models, as shown in Ref. [47] from a dynamical analysis in the Luo-Rudy model [48]. Quantitatively, however, the two models are quite different. At an 85% block of the I_Kr_ current, the ORd model displays EADs, whereas the TP06 model does not show any EADs even with a total block of I_Kr_ and still needs further reduction of RR by, e.g., increasing the conductance of I_CaL_. In this regard, the ORd model is consistent with the experiments conducted on human ventricular cells in Ref. [49]. We have investigated the rate dependence of the EADs in both models, and we find that they are quite different. At 85% block of I_Kr_ and higher, the ORd model shows EADs prominently at large PCL (slow rate dependence). This behavior is consistent with the experiments conducted with I_Kr_-blocking drugs like almokalant [11] and dofetilide [50]. In the TP06 model, EADs cannot be generated at any percentage block of I_Kr_ so the rate dependence of EADs cannot be studied here if we only block I_Kr_. However, when we reduce the RR even more by enhancing I_CaL_, the TP06 model diplays EADs at low PCL (fast rate dependence) and the ORd model continues to display slow rate dependence. It is known that EADs induced by drugs like isoproterenol, which affects both I_CaL_ (enhanced) and I_Kr_ (reduced), are fast rate dependent [11]. Therefore, in this regard, the TP06 model leads to results in conformity with experiments that use isoproterenol [11], which enchances I_CaL_ and reduces I_Kr_; however, we must exercise caution while making such inferences because isoproterenol also affects various other ion channels [21]. In future studies, it would be interesting to incorporate the detailed effects of drugs in both ORd and TP06 models and then to perform a systematic, comparative study of different drugs on EADs.

In the second part of our study, we have investigated the effects of EADs on wave propagation in ventricular tissue. Our extensive *in silico* studies of the two mathematical models for human ventricular tissue have helped us to elucidate the different types of waves and wave patterns that can arise as a result of EAD cells. In particular, we have found three qualitatively different types of waves, namely, Na-Ca-mediated waves, Ca-mediated waves, and phase waves, which develop in the simulation domain as we progressively decrease the repolarization reserve of the cells. Phase waves have not been found hitherto in the ORd model, although they have been reported in a study of EADs in the TP06 model [23]. We have provided a detailed comparison of these wave-types in these two mathematical models for cardiac tissue. Firstly, we find that the Na-Ca-mediated waves show different spatial patterns in these two models: in the ORd model we obtain short-lived spiral waves; by contrast, in the TP06 model we do not find any spiral waves that sustain themselves for one complete rotation period. Such types of waves, mediated both by I_Na_ and I_CaL_, have also been reported in earlier studies [35, 51]. Secondly, on reducing the RR, we observe waves mediated only by I_CaL_ (Ca-mediated type). The spatiotemporal evolutions of Ca-mediated waves in these two models are similar: in particular, both produce stable spiral-wave patterns, and their power spectra *E*(*ω*) exhibit a prominent peak at the rotation frequency of the spiral waves. The spiral wavelengths in the ORd model are, however, larger than those in the TP06 model. Thirdly, on reducing the RR, phase waves occur. These phase waves are slightly different in the ORd and TP06 models. In the ORd model we obtain spirals or damped wave-trains (see Figs 11 and 13), on the application of an asymmetric pulse, whereas, in the TP06 model, a variety of patterns are possible (Ref. [23]). Overall we find that, despite the difference in the rate dependence of EADs in the two models, the models produce, qualitatively, the same wave-types: Na-Ca-mediated, Ca-mediated, and phase waves.

We hope that our *in silico* confirmation of the existence of phase waves in a second, different, human-cell model (the ORd model) will lead to experimental studies of such waves in cell-culture experiments. Such experimental studies will also be able to build upon our detailed comparison of different wave types in ORd and TP06 models, with parameters that lead to EADs, and, thereby, help in the development of a detailed understanding of the propagation of different types of waves of electrical activation in cardiac tissue with cells that exhibit EAD.

We end with some of the limitations of our study. Our study concentrates on wave propagation in 2D simulation domains. We provide a few results for representative parameter sets in three-dimensional (3D) simulation domains for each wave-type. Clearly, our work has to be extended to anatomically realistic, 3D, simulation domains and we should account for the architecture of muscle fibers and their rotation. Furthermore, our study does not use a bidomain model, which takes into account the extra-cellular space [52]. We also have not incorporated the detailed effects of drugs, or modelled clinical settings to obtain EADs. Note, however, that our essential qualitative results for both 2D and 3D tissue simulations with EADs, e.g., the existence of Na-Ca- and Ca-mediated waves have been seen in experiments [35, 51].

## Author Contributions

Conceived and designed the experiments: SZ NV ARN AVP RP. Performed the experiments: SZ NV. Analyzed the data: SZ NV AVP ARN RP. Contributed reagents/materials/analysis tools: SZ NV ARN AVP RP. Wrote the paper: SZ NV RP.

## Supporting Information

S1 Video Spatiotemporal evolution of the three wave types. Video showing the spatiotemporal evolution of the transmembrane potential of the three types of waves, namely, Na-Ca-mediated (left panel), Ca-mediated (middle panel) and phase waves (right panel). The parameter sets are as follows. Na-Ca-mediated wave: G_CaL_ = 4 and G_kr_ = 0.37; Ca-mediated wave: G_CaL_ = 4 and G_kr_ = 0.275; phase wave: G_CaL_ = 4 and G_kr_ = 0.2. For the video, we use 10 frames per second with each frame separated from the succeeding frame by 20ms in real time.
(MPEG)

S2 Video Spatiotemporal evolution of the waves in the presence of obstacles. Video showing the spatiotemporal evolution of the transmembrane potential of the three wave-types, namely, Na-Ca-mediated, Ca-mediated, and phase waves, in the presence of a mesh of impenetrable obstacles. The parameter sets are as follows. Na-Ca-mediated wave: G_CaL_ = 4 and G_kr_ = 0.37; Ca-mediated wave: G_CaL_ = 2 and G_kr_ = 0.175; phase wave: G_CaL_ = 5 and G_kr_ = 0.225. For the video, we use 5 frames per second.
(MPEG)

S3 Video Na-Ca-mediated waves in our 2D simulation domain: A comparison of the Na-Ca-mediated wave type for the ORd and the TP06 models for representative parameter sets. ORd: G_CaL_ = 4 and G_kr_ = 0.37; TP06: G_CaL_ = 3 and G_kr_ = 0.5. For the video, we use 5 frames per second with each frame separated from the succeeding frame by 20ms in real time.
(MPEG)

S4 Video Ca-mediated waves in our 2D simulation domain: A comparison of the Ca-mediated wave type for the ORd and the TP06 models for representative parameter sets. ORd: G_CaL_ = 4 and G_kr_ = 0.275; TP06: G_CaL_ = 3.5 and G_kr_ = 0. For the video, we use 10 frames per second; in real time each frame is separated from the succeeding frame by 20ms.
(MPEG)

S5 Video Phase waves in our 2D simulation domain: A comparison of representative phase-wave patterns for the ORd and the TP06 models. The parameter sets are as follows. ORd: G_CaL_ = 4 and G_kr_ = 0.2; TP06: G_CaL_ = 4 and G_kr_ = 0. For the video, we use 10 frames per second with each frame separated from the succeeding frame by 20ms in real time.
(MPEG)

S6 Video Na-Ca-mediated waves in our 3D simulation domain: A comparison of the Na-Ca-mediated wave-type, in a 3D tissue, for the ORd and the TP06 models for representative parameter sets. ORd: G_CaL_ = 4 and G_kr_ = 0.37; TP06: G_CaL_ = 3 and G_kr_ = 0. For the video, we use 10 frames per second with each frame separated from the succeeding frame by 20ms in real time.
(MPEG)

S7 Video Ca-mediated waves in our 3D simulation domain: A comparison of the Ca-mediated wave-type, in a 3D tissue, for the ORd and the TP06 models for representative parameter sets. ORd: G_CaL_ = 4 and G_kr_ = 0.275; TP06: G_CaL_ = 4 and G_kr_ = 0. For the video, we use 10 frames per second with each frame separated from the succeeding frame by 20ms in real time.
(MPEG)

S8 Video Phase waves in our 3D simulation domain: A comparison of representative phase wave patterns, in a 3D tissue, for the ORd and the TP06 models. The parameter sets are as follows. ORd: G_CaL_ = 4 and G_kr_ = 0.2; TP06: G_CaL_ = 6.8 and G_kr_ = 0.1. For the video, we use 10 frames per second with each frame separated from the succeeding frame by 20ms in real time.
(MPEG)
